# Visual Presentation Effects on Identification of Multiple Environmental Sounds

**DOI:** 10.3389/fnint.2016.00011

**Published:** 2016-03-04

**Authors:** Yuko Masakura, Makoto Ichikawa, Koichi Shimono, Reio Nakatsuka

**Affiliations:** ^1^Faculty of Media Theories and Production, Aichi Shukutoku UniversityAichi, Japan; ^2^Faculty of Engineering, Yamaguchi UniversityYamaguchi, Japan; ^3^Department of Psychology, Chiba UniversityChiba, Japan; ^4^Faculty of Marine Technology, Tokyo University of Marine Science and TechnologyTokyo, Japan

**Keywords:** mixed sounds, visual stimulus content, visual stimulus timing, false hearing, visual representation

## Abstract

This study examined how the contents and timing of a visual stimulus affect the identification of mixed sounds recorded in a daily life environment. For experiments, we presented four environment sounds as auditory stimuli for 5 s along with a picture or a written word as a visual stimulus that might or might not denote the source of one of the four sounds. Three conditions of temporal relations between the visual stimuli and sounds were used. The visual stimulus was presented either: (a) for 5 s simultaneously with the sound; (b) for 5 s, 1 s before the sound (SOA between the audio and visual stimuli was 6 s); or (c) for 33 ms, 1 s before the sound (SOA was 1033 ms). Participants reported all identifiable sounds for those audio–visual stimuli. To characterize the effects of visual stimuli on sound identification, the following were used: the identification rates of sounds for which the visual stimulus denoted its sound source, the rates of other sounds for which the visual stimulus did not denote the sound source, and the frequency of false hearing of a sound that was not presented for each sound set. Results of the four experiments demonstrated that a picture or a written word promoted identification of the sound when it was related to the sound, particularly when the visual stimulus was presented for 5 s simultaneously with the sounds. However, a visual stimulus preceding the sounds had a benefit only for the picture, not for the written word. Furthermore, presentation with a picture denoting a sound simultaneously with the sound reduced the frequency of false hearing. These results suggest three ways that presenting a visual stimulus affects identification of the auditory stimulus. First, activation of the visual representation extracted directly from the picture promotes identification of the denoted sound and suppresses the processing of sounds for which the visual stimulus did not denote the sound source. Second, effects based on processing of the conceptual information promote identification of the denoted sound and suppress the processing of sounds for which the visual stimulus did not denote the sound source. Third, processing of the concurrent visual representation suppresses false hearing.

## Introduction

Many studies have been conducted to ascertain how auditory and visual modalities mutually interact. Many interactions have been examined only under conditions in which a participant is exposed to a single visual stimulus and a single auditory stimulus. In daily life, however, we are enveloped in and bombarded by multiple auditory and visual stimuli. Consequently, it is important to elucidate how the two modalities interact under the multiple-stimuli condition to reveal the processes used in daily life. This study specifically examines the interaction under the multiple-stimulus condition and particularly investigates how visual processing facilitates or interferes with auditory processing when the visual stimulus is relevant or irrelevant to the auditory stimuli.

Numerous previous reports have described that auditory processing interacts with visual processing in several ways. For instance, some studies have demonstrated that vision can dominate audition in determining perception of spatial aspects related to a stimulus. For example, the perceived location of a sound source tends to be shifted to the location of a visual stimulus (Stratton, 1897a,b; Young, 1928; Ewert, 1930; Willey et al., 1937; Thomas, 1941). This effect is known as *visual capture* (Jackson, 1953; Hay et al., 1965) or the *ventriloquism effect* (Howard and Templeton, 1966; Jack and Thurlow, 1973; Bertelson and Radeau, 1981). Different studies have demonstrated that audition can modify vision through duration perception (Walker and Scott, 1981), frequency perception (Welch et al., 1986; Shams et al., 2000, 2002; Wada et al., 2003; McCormick and Mammasian, 2008), and apparent motion (Kamitani and Shimojo, 2001; Wada et al., 2003; Ichikawa and Masakura, 2006). These results of studies suggest that the dominant modality in interaction between auditory and visual processing depends upon whether a participant judges the spatial or temporal aspect of the stimulus (Shimojo and Shams, 2001). Recent Bayesian models (e.g., Battaglia et al., 2003; Ernst, 2006) show that dominance of a modality in audio–visual interaction can be expected to result from experience related to the reliability of each modality in our daily life.

Previous studies examined audio–visual interaction using congruent and incongruent visual and auditory stimuli. For instance, studies of visual search have found that presenting characteristic sounds might enhance visual searching of corresponding objects, although presenting the name of the object as written word has no effect (Iordanescu et al., 2008). Studies of object recognition have found that presenting a semantically congruent sound might improve sound source identification (Chen and Spence, 2010). Presenting picture and sound elements that are mutually congruent, might hasten the recognition of objects that are denoted by the picture and sound, but presenting an incongruent picture and sound has no interference effect (Molholm et al., 2004). Studies of speech perception have revealed that congruent visual information might enhance target voice detection in a noisy environment (Sumby and Pollack, 1954; Campbell and Dodd, 1980; MacLeod and Summerfield, 1990; Thompson, 1995). A motion picture of a face speaking incongruent vowels is expected to modify pronunciation perception (McGurk and MacDonald, 1976; Sekiyama and Tohkura, 1993). In addition, a motion picture of a speaking face is expected to facilitate listening to the target sounds in terms of grouping of congruent motion pictures and sounds (Driver, 1996).

Interactions between vision and auditory processing can also be found in sound identification. Crossmodal priming studies, for example, have revealed that prior presentation of a picture hastened and improved identification of a sound when a picture was relevant to a sound, compared to when it was not (e.g., Greene et al., 2001; Noppeney et al., 2008; Schneider et al., 2008; Ozcan and van Egmond, 2009). A similar priming effect was found in auditory word recognition when a spoken word was presented after presentation of a written word corresponding to the spoken word (e.g., Holcomb and Anderson, 1993; Noppeney et al., 2008). These results of studies suggest that visual prime, irrespective of a picture or a written word, facilitates identification of the following relevant sound. However, from those earlier studies, it remains unclear how visual processing affects identification of the auditory stimulus when multiple auditory stimuli, relevant or irrelevant, are presented simultaneously as they are in daily life.

To elucidate how visual processing affects the identification of auditory stimuli, this study examined the effect of a visual stimulus on auditory processing with visual stimuli of different types and with various temporal relations between the visual and auditory stimuli in four experiments. To elucidate the effects of information obtained from the visual system on the identification of sounds, in the four experiments, we presented a picture or a written word which might or might not denote the source of one of four sounds recorded in a daily life environment. Then we examined how the visual representation of an object affects the auditory stimulus identification. Furthermore, we investigated the mechanism by which the time course of the visual–audio stimuli affects the visual–audio interaction. Manipulating the temporal relation between the audio and visual stimuli is expected to yield important information about what information processing is involved in crossmodal processing. For instance, we might infer that crossmodal effects, which are found only if the audio stimulus is presented concurrently with the visual stimulus, would be based on real-time perceptual processing. Furthermore, we might infer that the crossmodal effects which are found with a visual stimulus preceding an auditory stimulus, would be based upon the cognitive processing of the visual representation formed in a past observation of the visual stimulus. In addition, crossmodal effects, which are found with quite a short-term presentation of visual stimulus, would be based on the processing of the visual information obtained within a short period, such as low spatial frequency components (Schyns and Oliva, 1994). In fact, previous studies of the effects of presenting audio stimulus on the identification of visual stimulus (Chen and Spence, 2010, 2011) revealed that presenting a semantically congruent audio stimulus might facilitate the identification of the visual stimulus with various stimulus onset asynchronies (SOAs) between the audio and visual stimuli (less than 500 ms). This result indicates that facilitation of visual stimulus identification depends on real-time perceptual processing. However, few studies have examined the degree to which the temporal relation between the audio and visual stimuli affects the visual stimulus’ influence upon the audio stimulus. To elucidate the time course of visual–audio interaction, we manipulated the inter-stimulus interval between visual stimulus (a picture) and auditory stimuli in Experiments 1–3. In Experiment 1, the picture was presented simultaneously with the auditory stimulus. In Experiment 2, the picture was presented before the auditory stimulus. In Experiment 3, the picture was presented for an extremely short period before the auditory stimulus. In Experiment 4, we presented a written word as a visual stimulus, instead of a picture, under the same inter-stimulus conditions used in Experiments 1 and 2. The participant was instructed to identify all sounds involved in the auditory stimuli. We used the correct identification rate of the sounds that were presented, and the frequency of hearing the sound that was not presented (frequency of “false hearing”) as indexes of the effect of the visual stimulus. Comparison of the effects of a picture and written word on the identification of the auditory stimulus might reveal how either the visual representation or conceptual information derived from the visual stimulus affects the identification of the auditory stimulus. We also compare the effects of a temporal relation between the visual and auditory stimuli and discuss how information derived from a visual system is processed to identify the sounds in hearing multi-auditory stimuli.

## Experiment 1

In the first experiment, we presented a single visual stimulus and multiple auditory stimuli simultaneously to assess the effects of a visual stimulus on the identification of auditory stimuli. As auditory stimuli, we used natural sounds that are audible in our daily life and which are readily identified when presented separately. As the visual stimulus, we used a picture (static photograph), which might or might not correspond to what one of the auditory stimuli represented: the picture was relevant or irrelevant to the auditory stimulus. The durations of the visual and auditory stimuli were sufficiently long for participants to judge what were depicted and represented. Referring to the results of previous studies (Holcomb and Anderson, 1993; Greene et al., 2001; Noppeney et al., 2008; Schneider et al., 2008; Ozcan and van Egmond, 2009), we expected that presenting visual information for the auditory stimulus facilitates identification of the auditory stimulus.

### Methods

#### Participants

The experiment included 20 university students (4 females, 16 males) with ages of 20–25 years. All had normal hearing and normal or corrected-to-normal visual acuity. All were naïve to the purpose of this study. The experiments in this study were approved by the local ethical committee of the department of perceptual sciences and design engineering in Yamaguchi University.

#### Apparatus and Stimuli

A 21-inch display (21C-S11; Mitsubishi Electric Corp.), controlled using a personal computer (Macintosh G3; Apple Computer Inc.), and two loudspeakers (MU-S7; Sony Corp.) were used to present the visual stimulus and the auditory stimuli. The display was placed on a table at the height of the participant’s eye level and in the frontoparallel plane arranged at a viewing distance of 180 cm. The horizontal center of the display was placed at the intersection between the midsagittal plane and the frontoparallel plane. The right and left speakers on the floor were placed, respectively, 107.5 cm right and left of the intersection. The visual stimulus presented in the display was 10.2 by 13.5 degree. The sound pressure for the auditory stimuli was 35 dB (in L_Aeq_).

Stimulus sets of two types were used: experimental and additional sets. For the experimental stimulus sets, we used 13 sounds as auditory stimuli, which were recorded in a daily life environment, and 16 pictures of objects or creatures as visual stimuli, which were also taken in daily life (Table 1). We created nine sound combinations, each of which included four sounds selected from the 13 sounds. The number of the combined sounds in a sound combination was chosen based on results from a preliminary experiment in which we examined the difficulty of identifying multiple sounds. The relevant visual stimulus means that the picture denotes a sound source that 8 of 10 participants reported in the preliminary experiment in which we presented one sound and asked participants to report its sound source. In contrast to the relevant visual stimulus, the irrelevant visual stimulus means that the picture denotes no sound source. In Experiment 1, the sound sets were presented with a visual stimulus relevant to one of the four sounds or irrelevant to all of them, or they were presented without the visual stimulus.

**Table 1:** **Auditory and visual stimulus conditions**.

Auditory stimulus	Visual stimulus Relevant condition	Irrelevent condition	No visual stimulus condition
**Electronic piano**	Playing electronic piano	Ambulance	None (blank screen)
Drum
Railway crossing alarm
Construction drill

**Sawing timber**	Sawing timber	Horse	None
Typewriter
Cicada’s chirp
Murmur of a brook

**Ringing bell**	Bell	Rooster	None
Construction drill
Dog’s bark
Electronic piano

**Cutting vegetables on a cutting board**	Cutting vegetables on a cutting board	Deep frying of foods	None
Frog’s croak
Sawing timber
Rooster’s crow

**Railway crossing alarm**	Railway crossing	Bowling alley	None
Drum
Cicada’s chirp
Ringing bell

**Rooster’s crow**	Rooster	Bell	None
Electronic piano
Dog’s bark
Drum

**Typewriting**	Typewriter	Platform of a railway station	None
Construction drill
Railway crossing alarm
Ringing bell

**Murmur of a brook**	Stream	Beating drum	None
Frog’s croak
Cutting vegetables on a cutting board
Cicada’s chirp

**Frog’s croaking**	Frog	Cutting vegetables on a cutting board	None
Typewriter
Sawing timber
Ringing bell

*Bolds indicate the target sound in each sound combination*.

We prepared 12 additional stimulus sets that presented one, two, three, or four sounds that had been selected from five newly recorded sounds. We were concerned that participants would adhere to an answer to give the same numbers of sounds for all stimuli if the stimuli always presented the same number of sounds. To avoid such participant adherence, we changed the number of the sounds presented in the additional stimuli. The responses for the additional stimulus sets were not recorded. Therefore they were not analyzed in the following sections of this manuscript.

#### Procedures

The experimental stimulus sets have three conditions in which four sounds were presented simultaneously as auditory stimuli with or without a visual stimulus. In those stimuli, one sound was selected randomly as a target in each sound set and was designated as the *target sound*. Participants did not know which sound was the target in each sound set. In the relevant visual stimulus condition, a picture that denoted the target sound source was presented with the sounds. In the irrelevant visual stimulus condition, a picture that denoted no sound source of the four sounds was presented with the sounds. In the no visual stimulus condition, a blank screen was presented with the sounds. Table 1 presents a list of the target sounds and pictures in each of the relevant and irrelevant visual stimulus conditions.

Each participant conducted 39 trials in which nine experimental sets for each of the three stimulus conditions and 12 additional stimulus sets were presented in random order. In each trial, we presented the visual (or no-visual) and auditory stimuli for 5.0 s simultaneously. After the stimulus presentation, the participants listed sound sources of all the sounds that they identified in each sound combination on a paper. No time restriction or number restriction of answers was used for the participants to describe the sounds. They took at most 30 s in each trial.

### Results and Discussion

To analyze the reported sounds, first, three naïve participants, who were newly recruited, judged whether or not the reported sounds corresponded to the presented sounds in each trial. Second, we classified the judgments into three categories: identification of a target sound, identification of non-target sound, and false hearing. False hearing is used to describe a report of a sound that was not presented in the sound combinations. Third, we calculated the rate of the identification of target sounds and that of identification of non-target sounds for all judgments (i.e., all reported sounds), and counted the frequency of false hearing for each sound set. No number restriction of answers was used for the participants to describe the sounds: the number decrement (or increment) of false hearing does not mean the number increment (or decrement) of correct hearing.

**Figure 1:**
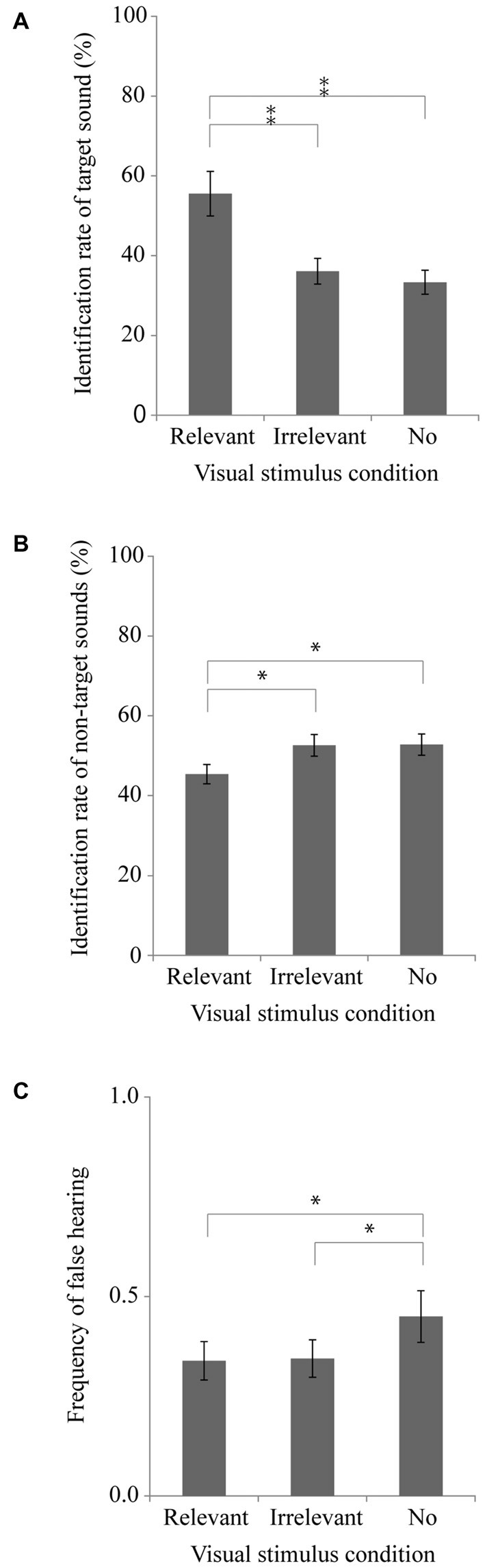
**Experiment 1 results.** Mean and SEM. **(A)** Target sound identification rate, **(B)** Non-target sound identification rate, and **(C)** False hearing frequency. The target sound identification rate and that of non-target sounds to all judgments (i.e., all reported sounds) were calculated for each visual stimulus condition. The false hearing frequency was counted for each sound. *p < 0.05, **p < 0.01.

Figures 1A,B respectively present the average rates of the identification of target sounds and of non-target sounds based on data from 20 participants for each visual stimulus condition. We conducted one-way repeated measures analyses of variance (ANOVA) with the visual stimulus condition, separately for the identification rate of target sound and that of non-target sound. The main effect of the visual stimulus condition was found to be significant [*F*_(2,38)_ = 14.60, *p* < 0.001, partial *η*^2^ = 0.43] for the target sound. Tukey’s *post hoc* HSD tests showed that the identification rate in the relevant visual stimulus condition was significantly higher than those in the other two visual stimulus conditions (*p* < 0.01). We also found a significant main effect [*F*_(2,38)_ = 10.99, *p* < 0.001, partial *η*^2^ = 0.37] for the non-target sound. Tukey’s HSD tests showed that the identification rate in the relevant visual stimulus condition was significantly lower than those in the other two visual stimulus conditions (*p* < 0.05). These results suggest that the visual image of the sound source promotes the identification of that sound, and that the visual image suppresses the identification of sounds for which the sound source was not presented. Details of the bases of these promotive and suppressive processes will be discussed in section “General Discussion”.

Figure 1C presents the mean false hearing frequency for each of the visual stimulus conditions. A one-way repeated measures ANOVA revealed a significant main effect of the visual stimulus condition for the frequency of false hearing [*F*_(2,38)_ = 3.42, *p* < 0.05, partial *η*^2^ = 0.15]. Tukey’s HSD tests showed that the frequency in the no visual stimulus condition was significantly higher than those in the other two visual stimulus conditions (*p* < 0.05). This result indicates that presentation of the irrelevant visual stimulus did not increase the false hearing, and that presenting a visual image of any object, irrespective of its relevance to the sounds, suppresses false hearing.

In Experiment 1, we found the facilitative effect of visual presentation on sounds identification, as we expected from the results of previous studies (Holcomb and Anderson, 1993; Greene et al., 2001; Noppeney et al., 2008; Schneider et al., 2008; Ozcan and van Egmond, 2009), and also found other effects. The results of Experiment 1 revealed that presentation of the visual image simultaneously with auditory stimulus has three effects on identification of the presented sounds: promotion of identification for the target sound, suppression of identification for the non-target sound, and false hearing. Two possible explanations exist for these effects. One is that these effects are based on perceptual processing in terms of the visual image presented simultaneously with sounds. Another is that these effects are based on cognitive processing in which visual representation of the past visual image affects the identification of the present sounds. In this case, the basis of the effects of visual image on identification of sounds is the priming effect of the visual representation, which is obtained shortly after the beginning of the visual stimulus presentation. This issue is examined further in Experiment 2.

## Experiment 2

Experiment 1 revealed the effects of presenting a visual image on identification of sounds presented simultaneously with the visual image. In the second experiment, we examine how cognitive processing, not the real-time perceptual processing, is involved in the effects of presenting visual image on identification of sounds. We presented the auditory stimuli after the presentation of the visual stimulus with ISI which was much longer than storage duration of visual iconic memory (Sperling, 1960).

### Methods

#### Participants

The second experiment included 20 newly recruited university students (4 females, 16 males) with ages of 20–25 years. All had normal hearing and normal or corrected-to-normal visual acuity. All were naïve to the purpose of this study.

#### Apparatus and Stimuli

Settings of the equipment and stimuli were identical to those used in Experiment 1. We used the same three conditions for the visual stimulus as those used in Experiment 1.

#### Procedures

Procedures were identical to those used in Experiment 1 except that the visual stimulus was presented for 5 s, 1 s before the auditory stimuli presentation (SOA between visual and auditory stimuli was 6.0 s).

### Results and Discussion

**Figure 2:**
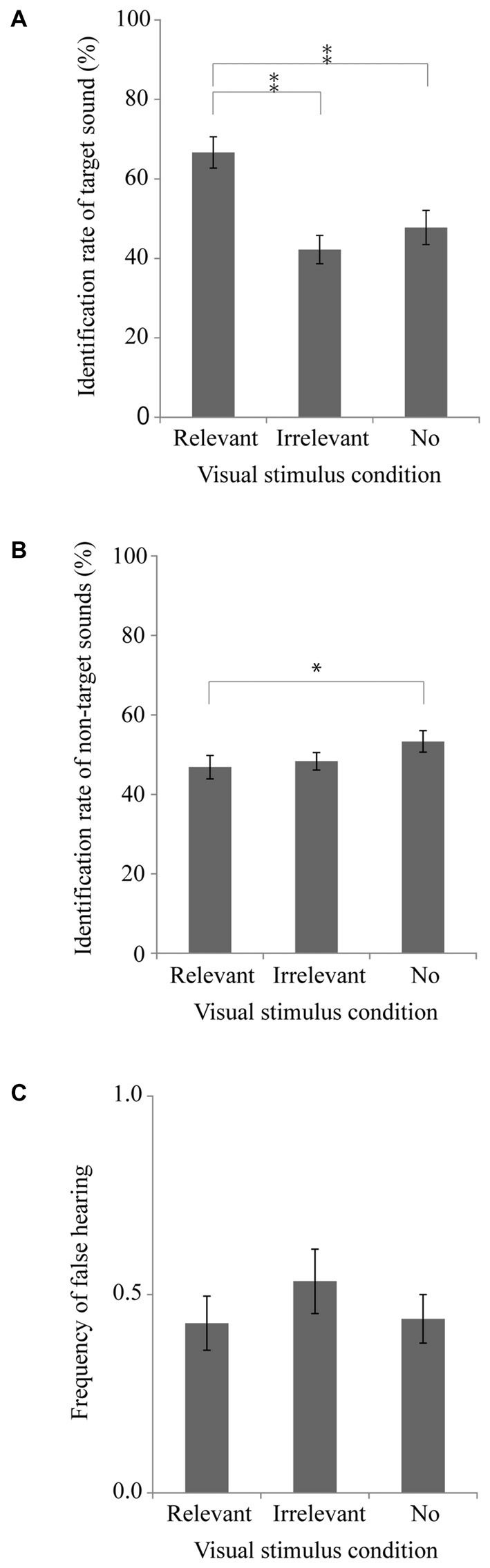
**Experiment 2 results.** Mean and SEM. **(A)** Target sound identification rate, **(B)** Non-target sound identification rate, and **(C)** False hearing frequency. *p < 0.05, **p < 0.01.

Figures 2A–C respectively portray the identification rate of the target sounds, the identification rate of the non-target sounds, and the frequency of false hearing. A one-way repeated measures ANOVA was conducted with the visual stimulus condition as a factor for the identification rate of the target sounds (Figure 2A). Results show a significant main effect of the visual stimulus condition [*F*_(2,38)_ = 14.84, *p* < 0.001, partial *η*^2^ = 0.44]. Tukey’s HSD tests showed that the identification rate in the relevant visual stimulus condition was significantly higher than those in the other two visual stimulus conditions (*p* < 0.01). This result suggests that presenting a visual image of the target sound affects promotion of the identification of that sound even when the visual stimulus precedes the auditory stimulus. We conducted the same one-way repeated measures ANOVA for the non-target sound identification rate (Figure 2B) and found a significant main effect [*F*_(2,38)_ = 4.90, *p* < 0.05, partial *η*^2^ = 0.21]. Tukey’s HSD tests showed that the identification rate in the relevant visual stimulus condition was significantly lower than that in the no visual stimulus condition (*p* < 0.05). However, we found no significant main effect of the visual stimulus condition in the frequency of false hearing [*F*_(2,38)_ = 2.71, *p* = 0.12, partial *η*^2^ = 0.13]. These results suggest that a visual image presented before sound sources including a target sound might promote identification of that sound, that it might suppress the identification of the non-target sounds in the sound sources, and that it might have no significant effect on false hearing for the visual image presented.

To examine differences in the effects of presenting visual images on sound identification between Experiments 1 and 2, we conducted two-way measures ANOVA with one between-subjects factor (i.e., timing of the visual presentation) and one within-subjects factor (i.e., visual stimulus conditions), separately for the identification rate of the target sound, that of the non-target, and false hearing. In analyzing the target identification rate, significant main effects were found for the timing of the visual presentation [*F*_(1,38)_ = 6.08, *p* < 0.05, partial *η*^2^ = 0.14] and for the visual stimulus condition [*F*_(2,76)_ = 28.61, *p* < 0.001, partial *η*^2^ = 0.43], although no significant interaction was found [*F*_(2,76)_ = 0.83, *p* = 0.38, partial *η*^2^ = 0.02]. The significant main effect of the timing indicates that the identification rate of the target sound in Experiment 2 is higher than that in Experiment 1 in either visual stimulus condition. These results suggest that presentation of a visual stimulus before an auditory stimulus might promote identification of the target sound in all visual stimulus conditions used. Tukey’s HSD tests of significant main effect for visual stimulus conditions demonstrated that the identification rate in the relevant visual stimulus condition was significantly higher than those in the other two visual stimulus conditions (*p* < 0.05), suggesting that a visual stimulus representing the target sound might promote the identification of that sound, irrespective of its timing for presentation.

In analyzing the identification rate of non-target sounds, a significant main effect was found for the visual stimulus condition [*F*_(2,76)_ = 12.39, *p* < 0.001, partial *η*^2^ = 0.25], although the main effect for the timing of the visual presentation was not significant [*F*_(1,38)_ = 0.05, *p* = 0.81, partial *η*^2^ = 0.00]. A tendency was apparent for the interaction of these two factors [*F*_(2,76)_ = 2.39, *p* = 0.09, partial *η*^2^ = 0.06]. Tukey’s HSD tests of the significant main effect for visual stimulus conditions showed that the identification rate in the relevant visual stimulus condition was significantly lower than that in the no visual stimulus condition (*p* < 0.05), suggesting that presenting a visual image of the sound source suppresses the identification of the other sounds, irrespective of the timing of the visual presentation.

Analysis of the false hearing frequency revealed significant interaction of the two factors [*F*_(2,76)_ = 4.19, *p* < 0.05, partial *η*^2^ = 0.10], although the main effects of the timing of the visual presentation [*F*_(1,38)_ = 1.26, *p* = 0.31, partial *η*^2^ = 0.03] and of the visual stimulus condition [*F*_(2,76)_ = 1.91, *p* = 0.26, partial *η*^2^ = 0.05] were not significant. Tukey’s HSD tests of the significant interaction showed that the false hearing frequency in the relevant visual stimulus condition was significantly lower than that in the no visual stimulus condition only when the visual stimulus was presented simultaneously with the auditory stimuli presentation (*p* < 0.05).

These results suggest that the effects of presentation of a visual image to promote the identification of the target sound and to suppress identification of the non-target sounds in the sound combination were not restricted to cases in which the visual stimulus was presented simultaneously with the auditory stimuli. These results imply that the effects depend upon cognitive processing in which the visual representation of the past visual image affects the present auditory identification. In contrast to these results, results for false hearing imply that the effect of presenting a visual image to suppress the false hearing was restricted to cases in which the visual stimulus was presented simultaneously with the auditory stimuli. This effect might be based upon perceptual processing of the visual and auditory stimuli, which were presented concurrently.

## Experiment 3

Experiments 1 and 2 showed that presenting a visual stimulus of the sound source had the effect of promoting identification of the target sounds and of suppressing the identification of the non-target sound, irrespective of the timing of the visual stimulus presentation. Many studies of priming effects have revealed that the visual stimulus presentation promotes identification of the sound even if the participant has difficulty understanding the visual stimulus contents (Noppeney et al., 2008). Experiment 3 investigated whether understanding of the visual stimulus contents affects promotion or suppression of sound identification. In the third experiment, we presented the auditory stimuli after the visual stimulus for, which was sufficient duration for visual processing to obtain low spatial frequency components from the image, but which was insufficient to obtain high spatial frequency components and color information (Schyns and Oliva, 1994).

### Methods

#### Participants

The third experiment included 20 newly recruited university students (8 females, 12 males) with ages of 19–23 years. All had normal hearing and normal or corrected-to-normal visual acuity. All were naïve to the purpose of this study.

#### Apparatus and Stimuli

The setting of the equipment and stimuli were identical to those in Experiment 1. We used the same three conditions for the visual stimulus as those used in Experiment 1.

#### Procedures

The procedures were identical to those used in Experiment 1, except that the visual stimulus was presented for 33 ms, 1 s before the auditory stimuli presentation (SOA between visual and auditory stimuli was 1033 ms). After the auditory stimuli presentation, the participants reported all the sounds that they identified in each sound combination in the same way as in Experiment 1.

To make sure that it was difficult for the participants to identify the visual stimulus for the short presentation time used in this experiment, we examined how correctly they reported what they saw in the preliminary test. The newly recruited 20 naïve participants reported what they saw. The rate of correct reports was 8.33%, which implies that most pictures used in this experiment were difficult to identify and that the presentation time was sufficiently short for this experiment.

### Results and Discussion

**Figure 3:**
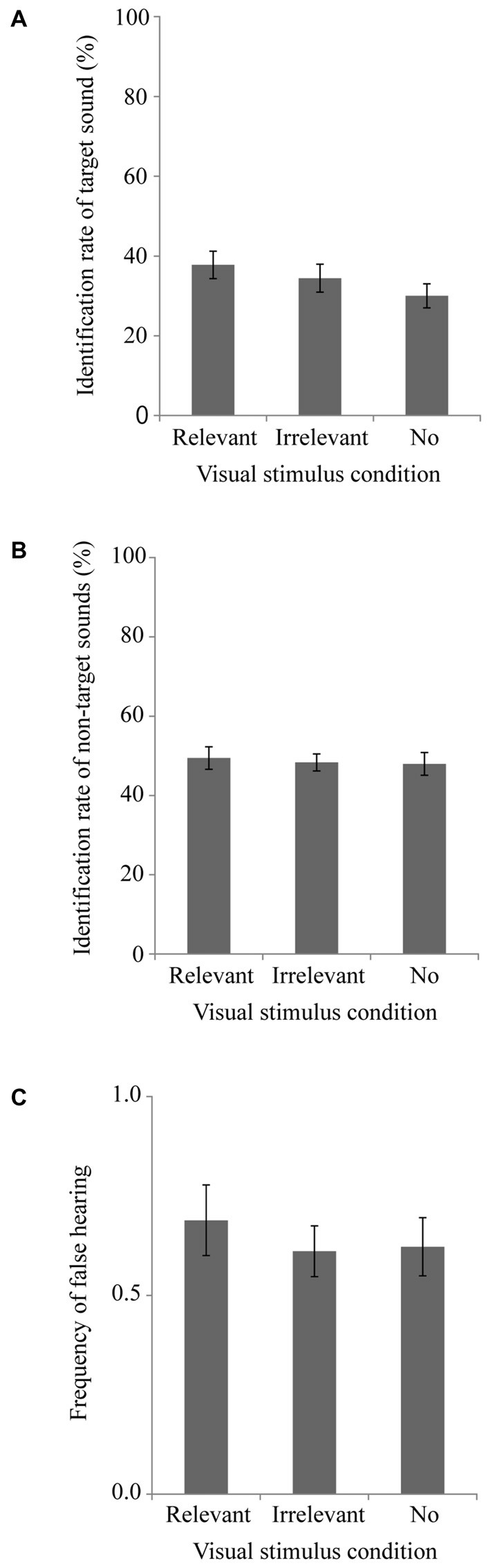
**Experiment 3 results.** Mean and SEM. **(A)** Target sound identification rate, **(B)** Non-target sound identification rate, and **(C)** False hearing frequency.

Figures 3A–C respectively portray the identification rate of the target sounds, the identification rate of the non-target sounds, and the frequency of false hearing. We conducted a one-way repeated measures ANOVA with the visual stimulus condition as a factor for the identification rate of the target sounds (Figure 3A). A tendency of the main effect of the visual stimulus condition was found [*F*_(2,38)_ = 2.78, *p* = 0.08, partial *η*^2^ = 0.13], but we were unable to find any significant main effect of the visual stimulus conditions for the non-target sound identification rate [*F*_(2,38)_ = 0.37, *p* = 0.69, partial *η*^2^ = 0.02] or the frequency of false hearing [*F*_(2,38)_ = 1.37, *p* = 0.27, partial *η*^2^ = 0.07]. These results suggest that the effect of presenting the visual stimulus to suppress the identification of the non-target sounds necessitates the duration that is sufficient for the participants to recognize what is depicted by the image. Even for the short presentation time, however, we can find a tendency of the main effect of the visual stimulus conditions. The duration of the stimulus presentation in this experiment was sufficient to obtain information of the low spatial frequency components in the image, but it was too short to obtain the information of the high spatial frequency components and colors (Schyns and Oliva, 1994). The present results suggest that the information of the low spatial frequency components in the image is sufficient to facilitate identification of the relevant sound.

## Experiment 4

In Experiment 4, we examine whether a written word that denotes the sound source as a visual stimulus can affect the sound identification. As discussed earlier, a visual prime, whether a picture or a written word, might facilitate the identification of the subsequent relevant sound (e.g., Holcomb and Anderson, 1993; Greene et al., 2001; Noppeney et al., 2008; Schneider et al., 2008; Ozcan and van Egmond, 2009). The question arises of whether or not the visual representation of the sound source contributes to identification of the target sound.

### Methods

#### Participants

The fourth experiment included 40 newly recruited university students, 20 of whom participated for the concurrent condition (8 females, 12 males, 20–25 years old) and 20 of whom participated for the preceding condition (9 females, 11 males, 20–25 years old). All had normal hearing and normal or corrected-to-normal visual acuity. All were naïve to the purpose of this study.

#### Apparatus and Stimuli

The setting of the equipment was identical to that for Experiment 1, except that we used a written word instead of a picture as a visual stimulus. The written word was shown with white characters presented on a black background on the display. The size of each character was 0.68 × 0.68 degree at most. The range of the character count was 1–12. We used the same three conditions for the visual stimulus as those in Experiment 1.

#### Procedures

We used two timing conditions (concurrent and preceding) for the visual stimulus presentation. In the concurrent condition, we presented visual and auditory stimuli simultaneously for 5.0 s as in Experiment 1. In the preceding condition, we presented the visual stimulus for 5 s, 1 s before the auditory stimuli presentation as in Experiment 2 (SOA between visual and auditory stimuli was 6.0 s). Durations of the visual and auditory stimuli were 5.0 s.

We presented the nine sound sets for each visual stimulus condition, and 12 additional stimuli in random order for either timing condition. After the stimulus presentation, the participants reported all the sounds that they identified in each sound combination in the same way as in Experiment 1.

### Results and Discussion

**Figure 4:**
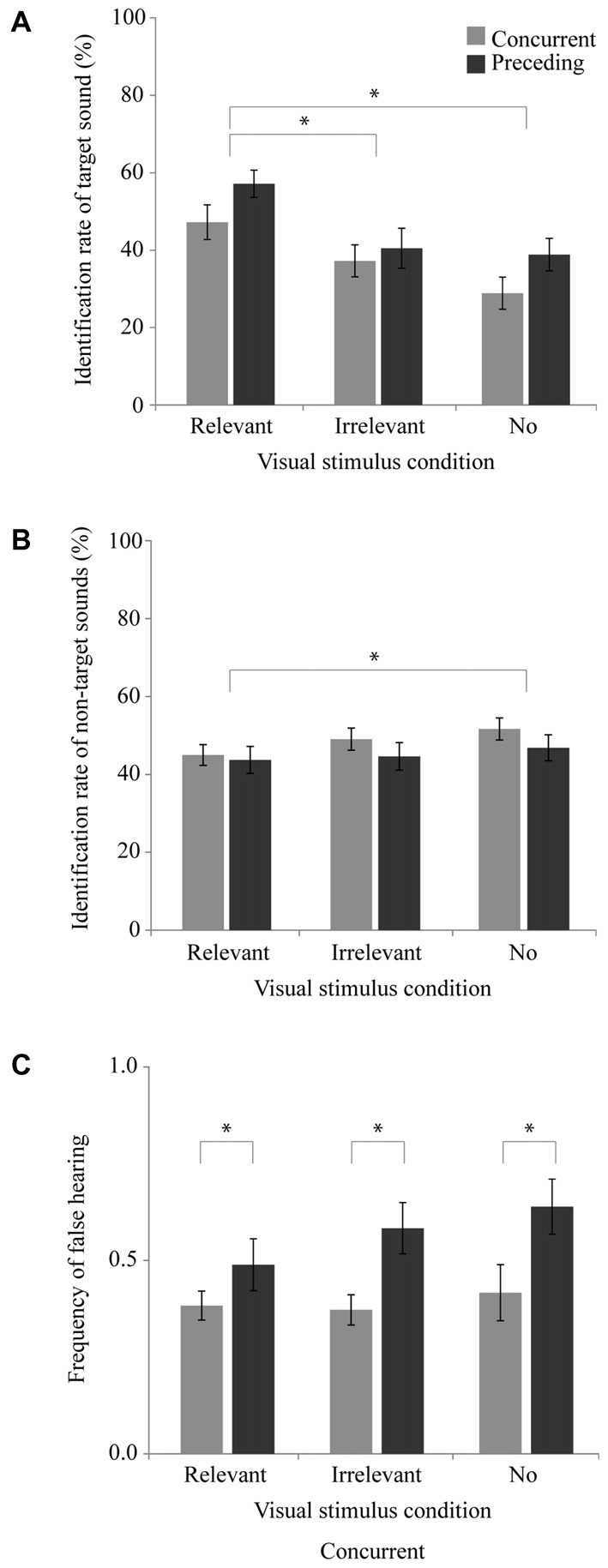
**Experiment 4 results.** Mean and SEM of the concurrent and preceding condition. **(A)** Target sound identification rate, **(B)** Non-target sound identification rate, and **(C)** False hearing frequency. *p < 0.05.

Figure 4 shows the respective identification rates of the target sounds (Figure 4A) and the non-target sounds (Figure 4B), as well as the false hearing frequency (Figure 4C). To examine the effect of word presentation and its timing on sounds identification, we conducted a two-way measures ANOVA with one between-subjects factor (i.e., timing condition) and one within subjects factor (i.e., the visual stimulus condition), separately for each of the identification rate of the target sound, that of the non-target sound, and the false hearing. In analyzing the identification rate of the target sound (Figure 4A), we found the main effect of the visual stimulus condition to be significant [*F*_(2,76)_ = 22.52, *p* < 0.001, partial *η*^2^ = 0.37], although the effect of the timing condition [*F*_(1,38)_ = 2.28, *p* = 0.10, partial *η*^2^ = 0.06] and the interaction of the two factors [*F*_(1,38)_ = 0.93, *p* = 0.40, partial *η*^2^ = 0.02] were not significant. Tukey’s HSD tests of significant main effect for visual stimulus condition showed that the identification rate in the relevant visual stimulus condition was significantly higher than those in the other two visual stimulus conditions (*p* < 0.05), suggesting that presenting the relevant written word to the target sound has the effect of promoting the identification of that sound. However, for identification of the target sound, no advantage was found when using the preceding presentation of the written word.

A two-way measures ANOVA for the identification of the non-target sound (Figure 4B) showed that the main effect of the visual stimulus condition was significant [*F*_(2,76)_ = 5.96, *p* < 0.01, partial *η*^2^ = 0.14], although neither the main effect of the timing condition [*F*_(1,38)_ = 0.66, *p* = 0.45, partial *η*^2^ = 0.02] nor interaction of the two factors [*F*_(2,76)_ = 0.92, *p* = 0.24, partial *η*^2^ = 0.02] was significant. Tukey’s HSD tests of significant main effect for visual stimulus condition showed that the identification rate in the relevant visual stimulus condition was significantly lower than that in the no visual stimulus condition (*p* < 0.05), suggesting that presenting a written word that denotes the sound source suppresses the identification of the other sounds.

A two-way measures ANOVA for the false hearing (Figure 4C) showed that the main effect of the timing condition was significant [*F*_(1,38)_ = 6.36, *p* < 0.05, partial *η*^2^ = 0.14], although neither the main effect of the visual stimulus condition [*F*_(2,76)_ = 2.44, *p* = 0.13, partial *η*^2^ = 0.06] nor the interaction of the two factors [*F*_(2,76)_ = 1.20, *p* = 0.33, partial *η*^2^ = 0.03] was significant. The significant main effect of the timing implies that the presentation of the written word precedent to the auditory stimuli would increase the false hearing frequency, rather than promoting the identification of the target sound. The result that the interaction of the two factors was not significant reveals that the effect of visual stimulus presentation to reduce the false hearing, which was found for presenting a picture (see “Experiment 2 Result” Section), was not clear for presenting a word.

## General Discussion

Four experiments demonstrated that presenting a visual stimulus has three effects on auditory identification under multiple sounds. First, presenting a picture or a written word promotes identification of the sound when its content is relevant to the sound. Second, presenting a picture or a written word representing the sounds suppresses the identification of the other sounds. Third, simultaneous presentation of a picture with the sounds, irrespective of its relevance to the sound, reduces false hearing: the participant heard sounds that were not presented in the sound combinations. In the following sections, these three effects are discussed.

As the first effect, both pictures and written words for which content denotes the target sound source promote identification of the target sound, particularly if the visual stimulus is presented for sufficient duration in four experiments. These results are compatible with the cross-modal priming effects on sound detection for both presenting a picture (e.g., Schneider et al., 2008; Ozcan and van Egmond, 2009) and a written word (e.g., Holcomb and Anderson, 1993; Greene et al., 2001) of the object generating that sound. These results suggest that both presenting the relevant picture and presenting the relevant written word can facilitate the identification of sounds in the sound combination. However, the benefit of the preceding presentation of the visual stimulus in the identification rate of the target sound was found only for the picture presentation, but not for the written word presentation. We assume that observing the picture activates the visual representation of the object which the picture represented, and that the visual representation extracted from the picture proactively promotes identification of the sound after the picture presentation. For the written word, from which we cannot extract the visual representation of the sound source directly, we were unable to find an advantage of the precedent presentation of the visual stimulus in Experiment 4. These results are compatible with the idea that activation of the visual representation contributes to identification of the corresponding auditory stimulus.

As the second effect, irrespective of the timing of the visual stimulus presentation, both the picture and written word for which contents denote the source of the target sound suppresses identification of the non-target sounds. The number of the sounds which participants identified in each trial were almost constant (*M* = 2.30, *SD* = 0.40 in Experiment 1), although three indexes of identification of a target sound, that of a non-target sound, and false hearing were not covariant because no restriction was made of the number of participants’ answers. It might be true that the participants shifted their criteria for reporting so that the number of answers was constant, and the identification of the target sound changed the response criteria not to report the non-target sound. Presenting a relevant visual stimulus with a sound source would enable participants to promote identification of a sound for which the sound source was presented. However, it would not enable participants to identify more sounds. Present results obtained for reduction of non-target sound identification in terms of presenting a picture and a written word demonstrated that the conceptual information of the sound source, rather than that visual representation, reduces the identification rate for the non-target sounds. The identification rate for the non-target sounds in Experiment 1 was similar to that in Experiment 2. The rate in Experiment 4, in which a written word was presented, was at a similar level irrespective of the timing for the visual stimulus. These results support the idea that the conceptual information related to the sound source reduces the identification rate for the non-target sounds. Some earlier studies (Holcomb and Anderson, 1993; Schneider et al., 2008; Ozcan and van Egmond, 2009; Vallet et al., 2010) have exaggerated the conceptual basis for the facilitating effects of presenting relevant picture on sound detection. The results reported herein suggest that the conceptual information has other effects on auditory processing, i.e., suppression of non-target sound detection.

As the third effect, the frequency of false hearing was reduced when a picture but not a written word was presented. This result demonstrates that presenting a picture of the sound source has the effect of suppressing the false identification of the sound that was not presented. This effect was not found for the preceding presentation of the picture. These results suggest that this effect of suppressing the false hearing is based not on the activation of the visual representation, which is the basis for the progressive promotion of the target sound identification, but on the concurrent presentation of the picture with the auditory stimuli. The concurrent presentation of the picture with the sound directs the perceptual processing toward a restricted area in the sound combinations.

We conducted four experiments to examine the effects of the content and timing of a visual stimulus on auditory processing. Results of these experiments imply that the visual/conceptual representation activated by concurrent presentation or prior presentation of the visual stimulus affects listening to sounds in at least three ways: (a) effects based on activation of the visual representation that is extracted directly from the picture, which promotes the identification of the denoted sound; (b) effects based on processing of the conceptual representation, which promotes the identification of the denoted sound, and suppress the processing of the irrelevant sounds; and (c) effects based on the processing of the concurrent visual representation, which suppresses false hearing. Experimental tasks used for this study were similar to our everyday tasks in a noisy environment. Therefore, one might expect that visual information affects our sound identification in our everyday life in terms of these three ways. As results of these experiments demonstrated, we propose that elucidating details of the bases of these three effects is effective for improving sound communication techniques in our everyday life using pictures and written words by promoting target sound identification and by suppressing false hearing.

## Author Contributions

YM: planning and conducting of experiments, writing of article. MI and KS: planning of experiments, writing of article. RN: planning and conducting of experiments. This study is supported by management expenses grants from Yamaguchi University to MI.

## Conflict of Interest Statement

The authors declare that the research was conducted in the absence of any commercial or financial relationships that could be construed as a potential conflict of interest.
